# Transcriptome alterations are enriched for synapse-associated genes in the striatum of subjects with obsessive-compulsive disorder

**DOI:** 10.1038/s41398-021-01290-1

**Published:** 2021-03-15

**Authors:** Sean C. Piantadosi, Lora L. McClain, Lambertus Klei, Jiebiao Wang, Brittany L. Chamberlain, Sara A. Springer, David A. Lewis, Bernie Devlin, Susanne E. Ahmari

**Affiliations:** 1 Center for Neuroscience, University of Pittsburgh, Pittsburgh, PA USA; 2 Department of Psychiatry, University of Pittsburgh, Pittsburgh, PA USA; 3 Department of Anesthesiology, University of Washington, Seattle, WA USA; 4 Department of Biostatistics, University of Pittsburgh, Pittsburgh, PA USA

**Keywords:** Molecular neuroscience, Psychiatric disorders, Pathogenesis

## Abstract

Obsessive-compulsive disorder (OCD) is a chronic and severe psychiatric disorder for which effective treatment options are limited. Structural and functional neuroimaging studies have consistently implicated the orbitofrontal cortex (OFC) and striatum in the pathophysiology of the disorder. Recent genetic evidence points to involvement of components of the excitatory synapse in the etiology of OCD. However, the transcriptional alterations that could link genetic risk to known structural and functional abnormalities remain mostly unknown. To assess potential transcriptional changes in the OFC and two striatal regions (caudate nucleus and nucleus accumbens) of OCD subjects relative to unaffected comparison subjects, we sequenced messenger RNA transcripts from these brain regions. In a joint analysis of all three regions, 904 transcripts were differentially expressed between 7 OCD versus 8 unaffected comparison subjects. Region-specific analyses highlighted a smaller number of differences, which concentrated in caudate and nucleus accumbens. Pathway analyses of the 904 differentially expressed transcripts showed enrichment for genes involved in synaptic signaling, with these synapse-associated genes displaying lower expression in OCD subjects relative to unaffected comparison subjects. Finally, we estimated that cell type fractions of medium spiny neurons were lower whereas vascular cells and astrocyte fractions were higher in tissue of OCD subjects. Together, these data provide the first unbiased examination of differentially expressed transcripts in both OFC and striatum of OCD subjects. These transcripts encoded synaptic proteins more often than expected by chance, and thus implicate the synapse as a vulnerable molecular compartment for OCD.

## Introduction

Obsessive-compulsive disorder (OCD), a chronic psychiatric illness, has a lifetime prevalence of 1–3%, affects approximately 50 million people worldwide, and can lead to significant impairment^1–3^. OCD is characterized by intrusive, recurrent thoughts (obsessions) and repetitive behaviors or mental acts (compulsions) that are often performed to reduce the anxiety associated with obsessions^4^. Even with current treatments, many patients continue to experience substantial symptoms and remission is rare^5^, underscoring the need for improved understanding of etiology.

Family^6,7^ and twin studies^7^ consistently support a genetic contribution to OCD. Heritability estimates of OCD, as well as obsessive-compulsive characteristics, indicate that genetic factors explain between 27% and 47% of the phenotypic variance^7–10^. Candidate gene association studies point to the glutamate transporter gene *SLC1A1*^11–15^ and the glutamate receptor genes *GRIN2B*^16,17^ and *GRIK2*^18–20^. Furthermore, genome-wide association studies (GWAS), though underpowered, converge on excitatory synaptic signaling as a potential source of dysfunction^21–23^.

Consistent with genetic evidence, neuroimaging research into the neural basis of OCD has highlighted dysfunction within the orbitofrontal cortex (OFC) and striatum, which are connected via dense cortico-striatal glutamatergic projections^4^. Both at rest and following symptom provocation, OCD subjects show hypermetabolism of glucose in the OFC relative to controls^24–27^, and increased blood-oxygen-level dependent signals in both OFC and caudate, a subregion of the dorsal striatum^28,29^. In addition, functional connectivity between OFC and striatum is increased in OCD subjects relative to healthy controls^30,31^ (though see ref. ^32^).

Causal links between excitatory synaptic dysfunction at cortico-striatal synapses and OCD-relevant behaviors have also been drawn from rodent studies. Mice with constitutive knockout of *Sapap3* (also known as *Dlgap3*), a post-synaptic density protein enriched at excitatory cortico-striatal synapses, or *Slitrk5*, a post-synaptic transmembrane protein found in excitatory synapses, display compulsive grooming phenotypes and cortical (*Slitrk5-KO*) and striatal (*Sapap3-KO*) hyperactivity that can be rescued with chronic fluoxetine (the first-line pharmacotherapy for OCD) treatment^33–35^. Furthermore, these models have altered expression of glutamate receptor transcripts within the striatum^33^, suggesting that removal of critical post-synaptic proteins affects both the structure and function of cortico-striatal synapses and may contribute to compulsive behavior.

Despite these links between genetic risk and neural alterations, and the supporting evidence of causal involvement provided by rodent studies, a dearth of information exists regarding molecular disruptions in these brain regions in OCD. To address this knowledge gap, we recently examined a targeted list of excitatory- and inhibitory-synapse-related transcripts in the OFC, caudate, and nucleus accumbens in OCD post-mortem brain tissue. This analysis demonstrated lower excitatory synapse-related transcripts in OCD subjects in both OFC and striatum, though reductions were more pronounced in the OFC^36^. However, this initial study only looked at a small subset of transcripts and therefore could not paint a complete picture of possible OFC and striatal dysfunction. Another recent study used unbiased RNA-sequencing (RNAseq) to identify gene expression differences in striatal subregions (caudate, putamen, and nucleus accumbens) of OCD subjects compared to unaffected comparison subjects, and observed differences in gene expression between striatal subregions. Genes that were differentially expressed between OCD subjects and unaffected comparison subjects in this study were also enriched for synaptic and immune function protein-encoding transcripts^37^. However, this study only examined the striatum and not its key glutamatergic input, the cortex. Here we used unbiased RNA sequencing to examine the transcriptome of two orbitofrontal cortex (medial and lateral OFC) and two striatal subregions (caudate and nucleus accumbens) in OCD subjects and unaffected comparison subjects.

## Methods

### Human Post-mortem Subjects

Brain specimens (*N* = 16) were obtained through the University of Pittsburgh Brain Tissue Donation Program during autopsies conducted by the Allegheny County Medical Examiner’s Office (Pittsburgh, PA) after consent was given by next-of-kin. An independent panel of experienced clinicians reviewed findings from structured interviews with family members, clinical records, toxicology, and neuropathology to make consensus DSM-IV diagnoses. Unaffected comparison subjects underwent identical assessments and were determined to be free of any lifetime psychiatric illnesses. All procedures were approved by the University of Pittsburgh’s Committee for the Oversight of Research and Clinical Training Involving Decedents and Institutional Review Board for Biomedical Research. To reduce biological variance between groups, each subject with OCD (*N* = 8) was matched with one unaffected comparison subject for sex (8 males and 8 females), age (standard error of the difference (SED = 0.29), post-mortem interval (PMI; SED = 3.18), and tissue pH (SED = 0.076). The age of unaffected comparison subjects ranged from 20 to 65 years, while the age of OCD subjects ranged from 20 to 69 years (Table 1).

**Table 1:** Cohort demographics.

	OCD subjects			Unaffected comparison subjects			df	*t*	*p*
	mean	SD	*n*	mean	SD	*n*			
Age (years)	47.4	14.70	7	47.6	13.42	8	13	0.03	0.98
pH	6.7	0.15	7	6.6	0.14	8	13	1.57	0.14
Post mortem interval (PMI; hours)	17.8	8.37	7	15.2	4.43	8	13	0.75	0.46
RNA ratio (260 nm/280 nm)	1.6	0.24	7	1.5	0.31	8	13	0.70	0.49
RNA integrity number (RIN)	7.8	0.44	7	7.6	0.65	8	13	0.46	0.65

*OCD* obsessive compulsive disorder, *PMI* post-mortem interval, *RIN* RNA integrity number, *SD* standard deviation, *n* count, *df* degrees of freedom, *t* Student’s *t*-statistic, *p* probability.

### Tissue collection and RNA extraction

Standardized amounts (50 mm^3^) of gray matter were collected from four separate brain regions– medial orbitofrontal cortex (mOFC, BA11), lateral orbitofrontal cortex (lOFC, BA47), head of the caudate nucleus, and nucleus accumbens core– and RNA was extracted as previously described^36^ (Details provided in Supplement 1).

### RNA sequencing

RNA sequencing and quality control (QC) steps are described in detail in Supplement 1. In brief, messenger RNA (mRNA) sequencing was performed at a targeted depth of 40 million reads per sample using the Illumina NextSeq 500 platform (Illumina Inc, San Diego, CA). mRNA reads (4 regions, 16 subjects) were mapped to 58,243 transcripts (Fig. S1), which were further reduced to 18,993 expressed RefSeq genes (v.2015-01). Of those, genes were retained that had at least 1 count per million in at least half of the samples for at least one brain region and if over all subjects and brain regions, gene j’s coefficient of variation CV_j_ < T, where T is defined as the mean of CV_j_ over all j plus 3 standard deviations of CV. This resulted in 14,211 genes whose expression passed QC. Next, we evaluated pairwise consensus correlations over genes. Correlation was high between BA11 and BA47, 0.71, compared to others (0.16–0.25; Fig. S2). Thus, data for BA11 and BA47 were treated as a single brain region termed “OFC” by averaging expression, per gene and subject, over BA11 and BA47. In this process, we noted one male OCD subject for whom the BA47 and nucleus accumbens samples had likely been inadvertently switched; this subject’s data were removed. After QC, 14,184 genes remained for analyses for all brain regions and 13,623, 13,889, and 13,756 for OFC, caudate, and NAcc, respectively.

### Differential expression

To identify drivers of variation in gene expression, the relationship between gene expression values and covariates—diagnosis, sex, age, post-mortem interval, pH, RNA integrity number, or brain region—were assessed using generalized linear regression^38^ (see Supplement 1 for details).

### Gene set enrichment

To investigate whether differentially expressed genes between OCD subjects and unaffected comparison subjects were enriched for biologically relevant gene-set pathways, we used the gene set enrichment analysis (GSEA) platform^39^. This platform contains various molecular signature databases (MSigDB, v7.0;^40^), including curated gene sets that are publicly available and biologically relevant. Enrichment was evaluated on the differentially expressed genes from the analysis of all brain regions together and per individual brain region (MSigDB sets used are given in Supplement 1).

### Cell type composition

The composition of the tissue samples in terms of 8 broad cell type fractions—excitatory neurons, medium spiny neurons (also known as spiny projection neurons), interneurons, astrocytes, oligodendrocytes, ependymal cells, immune cells, and vascular cells—was estimated using deconvolution methods^41^. The principle behind this method is that the bulk expression of a gene for a tissue sample, here measured using RNAseq, is a convolution of the number of cells of each type comprising the sample and the average expression of the gene within the cells of each type. Because medium spiny/spiny projection neurons are not found in the cerebral cortex and excitatory neurons are not found in the striatum, 7 cell types were analyzed for each of these regions and shared cell types were used for across-region averages. A detailed description of this procedure can be found in^41^ and in Supplement 1.

### Comparison with previous RNA-sequencing results

We also assessed agreement between our study and a recent study by Lisboa and colleagues^37^, which evaluated DEG in three striatal regions of six OCD and eight control subjects, by comparing DEG across the studies. After identifying a set of genes showing agreement between the two studies using the function ‘fdrThreshold’ (BioNet) to produce a *p*-value cutoff that corresponds to an FDR of 0.20 for the two striatal regions in our dataset, we determined gene set enrichment for one brain region, the caudate. We then analyzed enriched gene ontology (GO) terms using REVIGO^42^, which uses semantic similarity^43^ to cluster similar terms and prioritize terms for semantic interpretation by degree of enrichment. We used a similarity setting of 0.5, favoring a shorter and semantically diverse list of functions, and two default settings (semantic similarity measure SimRel and the database for GO term sizes “whole UniProt”). We used the online version of REVIGO (GO release “go_monthly-termdb.obo-xml.gz” [Jan 2017] and UniProt-to-GO mapping file “goa_uniprot_gcrp.gaf.gz” [15 Mar 2017]).

## Results

**Fig. 1:**
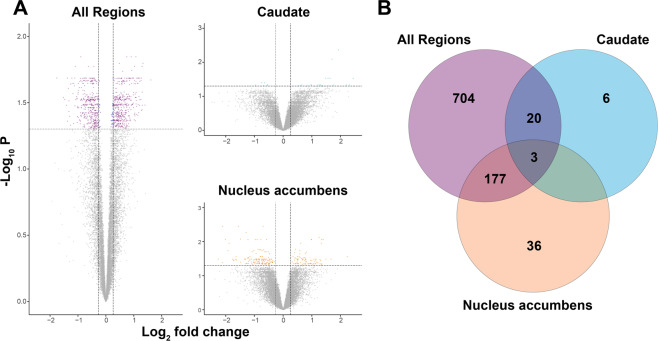
Volcano plots of differentially expressed genes between obsessive-compulsive disorder (OCD) subjects and unaffected comparison subjects. RNA sequencing was performed on post-mortem brain tissue originating from Brodmann areas 11 and 47 (OFC), the caudate, and the nucleus accumbens of 7 OCD subjects and 8 unaffected comparison subjects. **A** Left panel: differentially expressed genes were determined for all brain regions analyzed together. Upper right panel: differentially expressed genes in caudate. Lower right panel: differentially expressed genes in nucleus accumbens. The *y*-axis represents the (−log_10_*P*-value) and the x-axis represents the gene expression log_2_fold change. Vertical dashed lines (±0.26 log_2_ fold change) indicate gene expression differences between OCD subject and unaffected comparison subject cohorts, where upregulated genes are positive and downregulated genes are negative. The horizontal line demarcates significantly different gene expression differences between OCD subjects and unaffected comparison subjects (false discovery rate *q*-value <0.05; purple (≥0.26 or ≤ −0.26 log_2_ fold change)/blue (≤0.26 and ≥ −0.26 log_2_ fold change) dots = significant, gray dots = non-significant). Note: the volcano plot for the cortical regions (Brodmann areas 11 and 47) is shown in Fig. S3. **B** Venn diagram depicting the overlapping differentially expressed genes between all brain regions (purple), caudate (cyan), and nucleus accumbens (orange). There were no overlapping differentially expressed genes at the intersection between caudate and nucleus accumbens.

Here, we highlight two levels of analysis for differentially expressed genes (DEG): from three regions treated jointly (OFC, caudate, and nucleus accumbens), which we refer to as global analysis; and from each brain region considered separately, which we refer to as regional analysis. We first determined a parsimonious model for gene expression as a function of diagnosis (OCD versus unaffected comparison subjects) and other predictors. For the global analysis of DEG, predictors for the final model included diagnosis and brain region, as well as sex and pH as covariates (Table S1). It yielded 904 DEGs (FDR < 0.05; 471 were upregulated and 433 were downregulated). Predictors for the regional analyses were the same as the global analysis (e.g. diagnosis, with sex and pH as covariates), although effects were estimated by region. Between diagnostic groups, this analysis identified 29 (23 upregulated and 6 downregulated) and 216 (79 upregulated and 137 downregulated) DEG in the caudate and nucleus accumbens, respectively (FDR < 0.05; Fig. 1A; Table S2), and none for the OFC (Fig. S3). Examining overlap of DEG sets between global, caudate, and nucleus accumbens identifies 3 DEG (Fig. 1B). There were also 20 DEG in the intersection of the caudate and global gene sets and 177 DEG in the intersection of the nucleus accumbens and global gene sets (Fig. 1B). No overlapping DEGs were detected between the caudate and the nucleus accumbens alone.

### Gene set enrichment

DEG sets were next assessed for enrichment of gene functions (FDR adjusted for multiple testing, both here and for enrichment analysis). To do so, the 904 (global), 29 (caudate), and 216 (nucleus accumbens) DEG sets were queried against the MSigDB C2 curation, the C5 curation (GO sets), and the Hallmark gene sets. The global DEG were significantly enriched for 13 Reactome, 32 GO, and 5 Hallmark gene sets. DEG from the nucleus accumbens were enriched for one Hallmark gene set, and DEG from the caudate were enriched for one REACTOME and two GO gene sets after correction for multiple testing (Tables S3a/b).

**Fig. 2:**
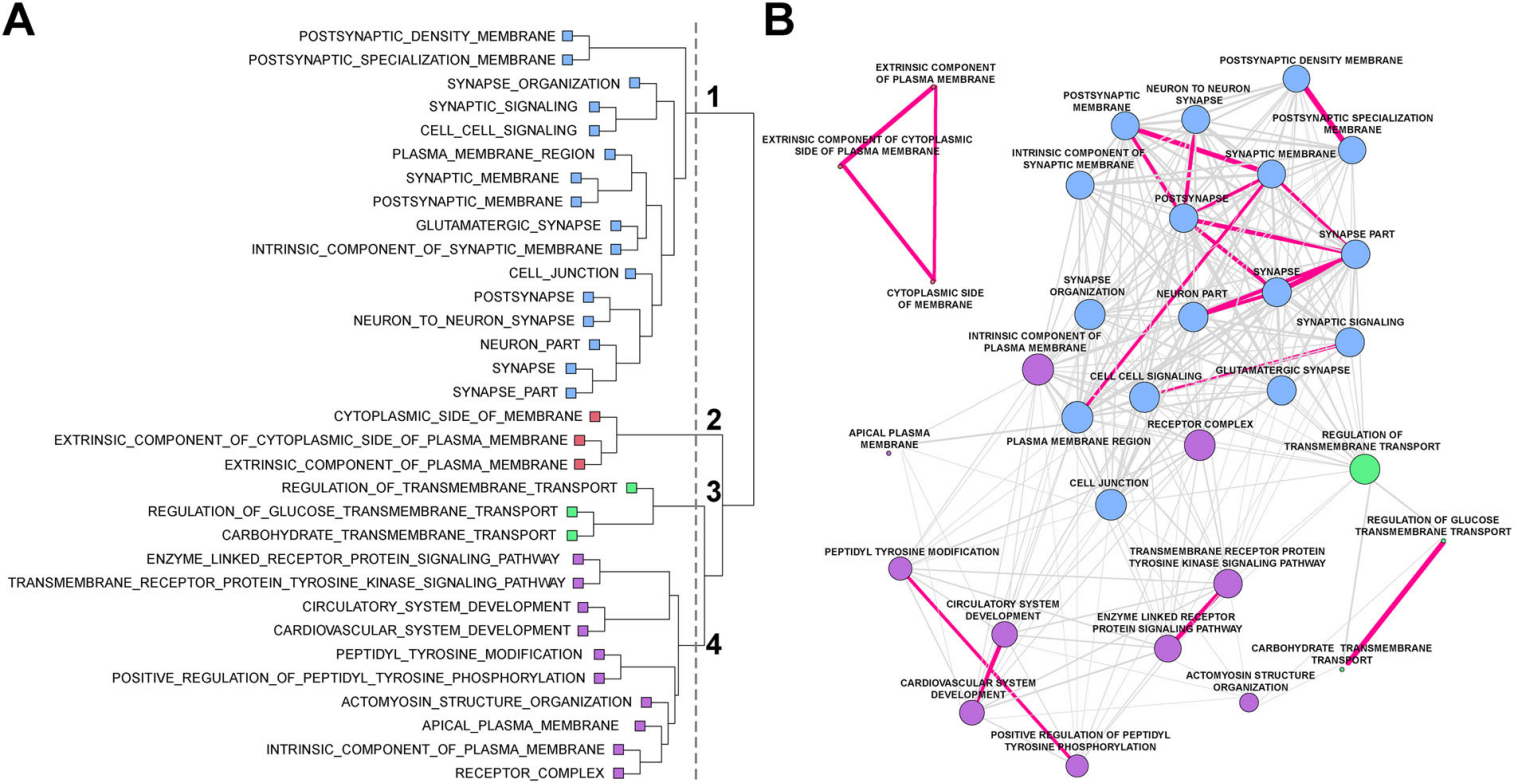
Gene set enrichment analysis for obsessive-compulsive disorder (OCD). Following RNA sequencing, global differentially expressed genes were determined between OCD subjects and unaffected comparison subjects (904 genes). Significant gene sets from the gene ontology (GO) pathways were determined using Fisher’s exact test. **A** Gene co-occurrence cluster dendrogram for gene set enrichment in OCD. Following gene set enrichment analysis, the number of co-occurring genes among the GO pathways were assessed. Four main branches were identified on the basis of the cut-point (vertical gray dashed line) established by visual inspection and color-coded per branch (blue: cluster 1; red: cluster 2, green: cluster 3, purple: cluster 4). **B** Cluster plot of gene sets associated with OCD. Genes were assessed using hierarchical clustering on the distances computed per gene set. The edges (lines) connecting nodes indicate the number of co-occurring OCD genes, where magenta lines have at least half of the genes co-occurring between the nodes (gene pathways), and gray lines indicate less than half of the OCD genes were co-occurring. Co-occurring genes were defined as the fraction of genes present in two pathways given the total number of unique genes represented in both pathways. The thickness of the magenta lines indicates more (thicker) or less (thinner) gene co-occurrence between two given gene sets. Note, the length of connecting vertices does not indicate distance between nodes.

For the global analysis, we noted that several of the enriched GO gene set pathways had a high proportion of overlapping genes. We leveraged this overlap to explore the links between enriched gene sets and draw broader conclusions about the nature of transcription alterations in OCD. Hierarchical clustering revealed four distinct sub-networks (clusters) of gene sets (Fig. 2). Of note, cluster 1 contained 16 gene sets that could be attributed to the synapse (Fig. 2). We also tabulated the number of enriched gene sets with either upregulated or downregulated DEG in OCD subjects versus unaffected comparison subjects (Table S3). Because our previous qPCR work^36^ identified lower levels of several synaptic transcripts within the OFC and striatum of OCD subjects compared to unaffected comparison subjects, we sought to determine whether the genes identified in these synaptic gene sets contained more downregulated genes than other clusters. For the 16 synaptic gene sets identified in cluster 1, the fraction of genes that were downregulated in each set was significantly higher than that for the remaining 16 gene sets (mean fraction of downregulated genes was 0.712 and 0.507, respectively; *t* = 6.26; df = 30; *p* = 6.7 × 10^−7^). Furthermore, we found that there were significant differences in the mean fraction of downregulated genes across the four clusters (analysis of variance, *F*[3,28] = 28.46, *p* = 1.19 × 10^−8^). The mean fraction of downregulated genes for clusters 1, 2, 3, and 4 was 0.76, 0.63, 0.69, and 0.38, respectively (Fig. 2; Table S3): across all genes contained in cluster 1, there was a significantly higher fraction of downregulated than upregulated genes (*t* = 4.91, Bonferroni corrected *p* = 1.34 × 10^−4^), while cluster 4, containing pathways involved in receptor tyrosine kinase activity, circulatory system development, and plasma membrane regulation, had a significantly higher fraction of upregulated genes (*t* = −7.4, Bonferroni corrected *p* = 4.94 × 10^−7^). Clusters 2 and 3, representing cytosolic plasma membrane components and transmembrane transport, respectively, had more similar levels of up and downregulated genes.

### Cell-type representation within tissue

**Fig. 3:**
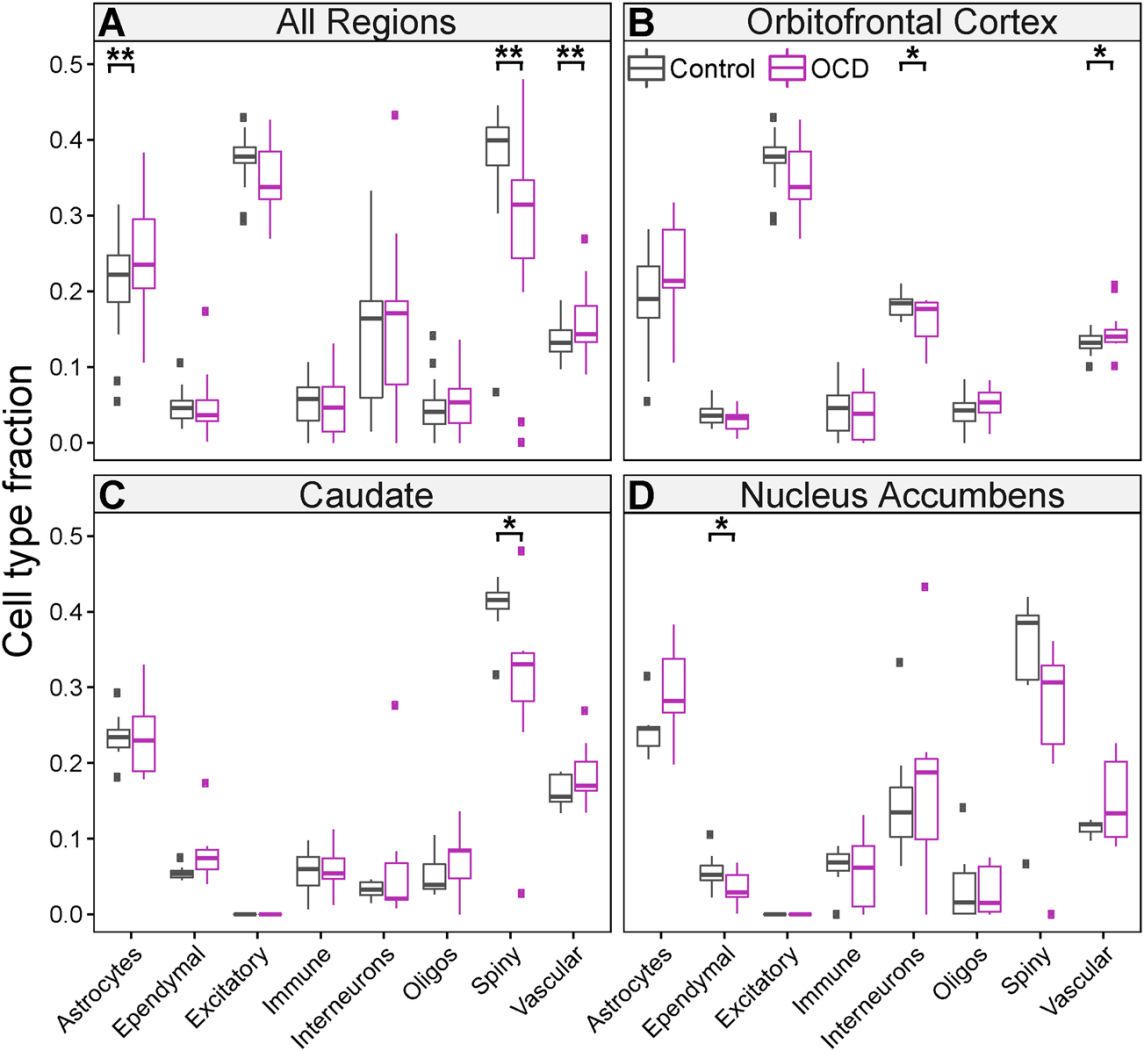
Cell type fractions in OCD subjects and unaffected comparison subjects. Cell type fractions were determined across all brain regions for OCD subjects (purple) and unaffected comparison subjects (grey) separately. Gene expression patterns in single-cell data were used to estimate the fraction of eight broadly defined cell types: astrocytes, ependymal cells, excitatory neurons, immune cells, interneurons, oligodendrocytes, medium spiny neurons (a.k.a. spiny projection neurons), and vascular cells. **A** Cell type fractions for global analysis (combined OFC, caudate, and nucleus accumbens). **B** Cell type fractions in the OFC, **C** Caudate, and **D** Nucleus accumbens. Boxplots are displayed, where ** indicates *p* < 0.01 and * indicates *p* < 0.05 (uncorrected for multiple comparisons). The mean cell type fractions were computed based on all four brain regions; however, when the cell type did not exist in the brain region, it was not included in the calculation. Thus, the mean cell type fraction for the excitatory neurons was based only on the cortical regions and the mean cell type fraction for spiny projection neurons was based on the caudate and nucleus accumbens regions.

We used the gene expression patterns in the bulk tissue for each of the three brain regions to estimate the fractions of eight broad cell types: excitatory neurons, inhibitory interneurons, medium spiny neurons (a.k.a. spiny projection neurons), astrocytes, ependymal cells, immune cells, oligodendrocytes, and vascular cells (Table S4). After adjusting for covariates, OCD subjects were significantly different from unaffected comparison subjects for the representation of the following cell types in the global analysis: astrocytes (*p* = 0.005), vascular cells (*p* = 0.002), and medium spiny neurons (*p* = 0.009; Fig. 3A).The remaining cell types were not significantly different. Astrocyte cell fractions were larger in OCD subject samples, as were vascular cell fractions, whereas medium spiny neuron fractions were smaller in OCD samples (Fig. 3A). Regional analyses suggest smaller interneuron fractions in the OFC (Fig. 3B), smaller medium spiny neuron fractions in the caudate (Fig. 3C), smaller ependymal cell fractions in the nucleus accumbens (Fig. 3D), and a larger fraction of vascular cells in the OFC of OCD subjects compared to unaffected comparison subjects (Fig. 3B; Table S5). While regional results do not achieve significance after correction for multiple testing, they are consistent with global results. Similar results were obtained using human single nucleus RNA (snRNA) sequencing as our reference dataset^44^ for deconvolution (Fig. S4 and Tables S6, S7), with an overall reduction in medium spiny neuron fractions (*p* = 0.001) and an increase in astrocytic fractions (*p* = 0.01) in the global analysis.

### Comparison with prior OCD RNA-sequencing study

A recent study by Lisboa and colleagues^37^ also evaluated DEG in three striatal brain regions from six OCD and eight control subjects. Since two of our study’s brain regions overlapped with theirs (caudate and nucleus accumbens), we compared the DEG sets. Because DEG provided by Lisboa et al. were only a subset of all genes they evaluated (specifically those having an uncorrected *p*-value <0.01), we evaluated this DEG set and limited our comparison to Ensembl genes. Matching the Lisboa DEG set with results from all transcripts detected in our study (Table S8) produced 541 and 126 genes overlapping across studies for caudate and nucleus accumbens, respectively (Table S9). Despite the relatively small sample size for each study, the correlations of their differential expression in log2 fold change were both substantial: 0.42 (caudate: *p* < 4.4 × 10^−25^) and 0.26 (NAc: *p* < 0.0032) (Table S8).

We next evaluated what fraction of the Lisboa DEG showed evidence for differential expression in our study in two ways. First, we fitted a beta-uniform mixture model as implemented in function “fitBumModel” in R library BioNet to the *p*-values from our study for the overlapping genes. For this model, the goal is to estimate the fraction of genes in our study inferred to have signal, given that they were DEG in the Lisboa study; i.e., what fraction of observations were drawn from the Beta distribution (Fig. S5). This estimate was 1.0, suggesting that all DEG from the Lisboa study carry signal in our data. In order to further validate the similarity between study results, we next used the function “fdrThreshold” (BioNet) to produce a *p*-value cutoff that corresponds to an FDR of 0.20 for both regions in our dataset. Test statistics for 310 and 71 genes fell below this FDR threshold for caudate and nucleus accumbens, respectively. Of these, 263 and 64 were congruent in the direction of their log fold change across studies (Fig. S5). Using the set of 263 genes from the caudate (i.e., those that were FDR ≤ 0.20 and congruent), we then compared enrichment in this gene set to enrichment found in the 13,348 protein-coding genes we analyzed for DEG that were not found in Lisboa’s DEG set. This analysis yielded 144 enriched gene sets (FDR < 0.05, Table S10), of which 115 were gene ontology or GO terms. We next analyzed these GO terms and their *q*-values for enrichment using REVIGO to produce a semantic interpretation of these results. Complementing our enrichment analysis of global DEG, the GO enrichment analysis of concordant genes across studies prominently highlights synaptic function (Fig. S6).

## Discussion

Although the etiology of OCD remains unknown, convergent evidence suggests disruptions in critical components of cortico-striatal glutamatergic synapses^7,21,22,36,37,45^. However, no comprehensive analysis of the OCD transcriptome has been performed simultaneously in tissue from OFC and striatum, the most commonly identified regions with functional and structural alterations in neuroimaging studies^31,46–50^. We therefore examined the transcriptome in two orbitofrontal (medial, lateral) and two striatal (caudate, nucleus accumbens) brain regions in subjects with OCD and unaffected comparison subjects. We first looked at global differential expression in all regions jointly and identified 904 transcripts that were differentially expressed as a function of OCD diagnosis (Fig. 1A, left). Interestingly, most DEG were found in the caudate and nucleus accumbens (Fig. 1A, right) and not in OFC (Fig. S3). Gene set enrichment and hierarchical clustering of all differentially expressed transcripts identified a hub of pathways involved in synaptic neurotransmission and associated with the glutamatergic synapse (Fig. 2). Together, these data further support the involvement of cortical and striatal glutamatergic synaptic dysfunction in OCD pathogenesis. Using our global DEG data, we also estimated broad cell type representation from our tissue samples, finding significantly different fractions of astrocytes, vascular cells, and medium spiny neurons between OCD and unaffected comparison subjects (Fig. 3).

Correlational evidence of structural and functional changes in both the OFC and striatum in OCD has accumulated over the past 30 years^4^. More recent rodent studies have functionally linked these regions to OCD-relevant behavior^51^ and highlighted a critical role for excitatory synaptic signaling through the use of transgenic knockout mice lacking proteins involved in normal excitatory synaptic neurotransmission^33–35,52,53^. Furthermore, genetic studies of OCD subjects frequently identify associations with genes involved in excitatory synaptic signaling^11,12,16,17,19–22,54^. Consistent with these findings, we observed alterations of transcripts involved in synaptic signaling in OCD subjects via gene-set analysis. Of the 32 significant gene-sets we identified, 16 were classified as involved in synaptic neurotransmission (Fig. 2). Genes in these sets were disproportionately downregulated (Table S3b), consistent with our previous qPCR findings in the tissue from these same subjects^36^ and with genetic knockout mice that display compulsive behavior^33,35^. Future molecular studies must investigate how downregulation of synaptic gene expression could lead to observed hyperactivity in the OFC and striatum of OCD patients. In the OFC, hyperactivity may be driven through a decrease in expression patterns representative of inhibitory interneurons (Fig. 3B), which regulate excitatory/inhibitory balance. In the striatum, downregulation of synaptic gene expression is likely related to a decrease in the expression profile of medium spiny neurons (Fig. 3A, C, D), which are the primary recipients of cortico-striatal glutamatergic input. It will be of interest to determine whether alterations in cell type expression profiles are due to a change in the absolute number of that cell type in OCD subjects, a shift in an individual cell’s expression profile (e.g., a decrease in the number of synapses), or compensatory changes due to alterations in other cell types.

In addition to strengthening the link between OCD pathophysiology and synaptic dysfunction in cortical and striatal brain regions, RNAseq and enrichment analysis allowed us to identify novel signaling pathways that may be disrupted in OCD subjects (Fig. 2). We find that gene sets related to cytosolic membrane components (cluster 2) and transmembrane transport (cluster 3) contain similar numbers of up and downregulated gene sets, suggesting complex differential regulation between OCD and unaffected comparison subjects. Cluster 2 pathways are comprised of genes encoding cytosolic proteins associated with receptors, such as G proteins and regulatory subunits, as well as other cytosolic components that are critical for intracellular signaling and interact with proteins found in cluster 1. Cluster 3 contains genes that regulate glucose and carbohydrate transport and may indicate different metabolic needs in the OFC and striatum of OCD subjects. This would be consistent with human imaging studies finding hyperactivity and increased glucose metabolism in these regions in OCD^24–26^, and could be related to the changes in cell type expression profiles observed in our cell type deconvolution (Fig. 3). For example, the metabolic needs of astrocytes, whose expression profile is increased in OCD (Fig. 3A), differ from that of neurons^55^.

Compared to synaptic enrichment (cluster 1), which included mostly downregulated DEG, cluster 4 pathways associated with circulatory and cardiovascular system development, transmembrane tyrosine kinase activity (RTK), and components of the plasma membrane, mostly consisted of upregulated DEG (Table S3). These upregulated gene sets are consistent with the increased vascular cell expression profile observed in our cell type deconvolution (Fig. 3A), and increased angiogenesis in these regions may play a role in the elevated blood-oxygen level dependent (BOLD) signal observed in functional imaging studies of OCD subjects. While previous studies of RTK alterations in OCD have identified an association between tropomyosin receptor kinase B/neurotrophic tyrosine kinase receptor type 2 (*TRKB/NTRK2*) and OCD^54^, our data suggest a role for other kinases, including Janus kinase 3 (*JAK3*) and protein kinase C beta (*PRKCB1*), though their precise functions remain to be determined.

To date, there remains a dearth of studies using an unbiased approach to examine the molecular underpinnings of OCD and related disorders, likely due to the difficulty of obtaining high quality post-mortem tissue samples. In the first transcriptomic study of OCD tissue, Jaffe and colleagues^56^ used a microarray approach to examine DEG in the dlPFC as a function of several disorders typified by obsessive thoughts and compulsive behaviors (eating disorders, OCD, OCPD, and tic disorder). The authors found 286 DEG when comparing OCD/OCPD/tic cases to controls. Interestingly, out of the 904 global DEG in our study, only 13 were identified as differentially expressed by Jaffe et al.^56^. While this is consistent with statistical challenges owing to the relatively small sample size for both studies (see^57^), it could also indicate that molecular dysregulation in the dlPFC could be distinct from what is seen in the adjacent OFC, as well as the striatum. This is especially interesting in light of neuroimaging findings that suggest hyperactivity at rest in the OFC^24,25,58,59^, while no such evidence exists for the dlPFC (though lateral OFC and dlPFC hypoactivity have been consistently observed in OCD patients during cognitive tasks^60,61^). Studies directly comparing differential expression in the dlPFC and OFC of the same subjects may clarify whether gene expression changes are global or region specific.

A second recent study by Lisboa and colleagues^37^ used RNAseq to assess gene expression in three striatal regions (caudate, putamen, and nucleus accumbens) in OCD and comparison subjects^37^. Our findings of differences in gene expression within striatal subregions, with no genes identified as differentially expressed in both the caudate and the nucleus accumbens (Fig. 1B), are broadly consistent with this work. Furthermore, direct comparison of genes identified as DE by Lisboa and colleagues and in our study identified 263 genes in the caudate and 64 genes in the nucleus accumbens that differed in the same direction as a function of OCD diagnosis (Fig. S5 and Table S8–9). Lisboa et al. also identified gene set enrichment of synaptic-related transcripts, supporting our observation of functional enrichment of highly correlated synaptic gene sets (Fig. 2; Table S3a) that could be primarily driven by changes in striatal gene expression. Similarly, we observed enrichment of synaptic transcripts when looking specifically at genes in the caudate that differed in the same direction between subject groups in the two studies, (Fig. S6 and Table S10). A key difference between our findings and those of Lisboa et al. is that our analyses did not reveal any group difference in immune- or microglia-related gene sets. There are many possible explanations for this, including the age of subjects (age-range 58–98 in Lisboa et al.^37^, age-range 20–69 in the current work), as aging is associated with changes in immune system gene expression^62^. The role of the immune system in OCD (and its interaction with age and disease length) requires further study.

Because post-mortem studies of psychiatric disease have typically relied on whole tissue homogenates^63^, they have traditionally been agnostic about the potential contributions of genetically and phenotypically distinct cell types to disease pathology. Here we addressed this limitation by using a deconvolutional approach to estimate cell type-specific fractions of whole tissue homogenates from OCD subjects and unaffected comparison subjects. We performed this analysis both using mouse single cell RNA-sequencing (scRNAseq) (Fig. 3) and human single nucleus RNA-sequencing (snRNAseq) (Fig. S4) data obtained from the striatum and cortex to generate signature matrices; both approaches generated similar conclusions. Here we present the data from the mouse signature matrix in the main text because nuclear RNA only accounts for 20–50% of RNA molecules in a neuron^64^, and more genes were reliably sampled from the scRNA sequencing experiment. Importantly, this analysis does not differentiate between differences in cell type-specific fractions due to changes in absolute cell number, or due to changes in the transcriptional activity of single cells. We do observe that OCD subjects have lower cell type expression profiles of medium spiny neurons, the predominant cell type in the striatum^65^, pointing us to future studies to discriminate between these two possibilities. Interestingly, we also observed increases in astrocyte and vascular cell type fractions in OCD subjects. Though there is limited evidence linking these cell types to OCD, it was recently demonstrated that altering astrocytic calcium-dependent signaling in mice produces an OCD-like compulsive grooming phenotype and striatal microcircuit deficits^66^. Furthermore, astrocytes and vascular cells interact at tight junctions to regulate blood-brain barrier function. An increase in these cell fractions might reflect a compensatory change due to the hypermetabolism commonly observed in both the OFC and striatum of OCD subjects^25,67–69^.

It is important to note several limitations of the present study and the measures we have taken to account for them. First, our cohort is small, containing only seven OCD subjects and eight unaffected comparison subjects. Obtaining post-mortem tissue with sufficiently high RNA quality to perform robust RNA-sequencing (Table 1; Fig. S1) remains a challenge, and because of this it is difficult to evaluate the effect of all potential covariates on gene expression. Here we built a model including only covariates that statistically affected transcript expression (see Methods). A second related point is that DEG may be detected due to several factors, including (1) large OCD versus unaffected subject differences over all three regions, (2) large differences within only one region, (3) or modest but consistent differences for all three regions, among other scenarios. Global analysis could detect DEG for any of these scenarios, within the limits of statistical power, whereas regional analysis would likely detect DEG only for (1) or (2). If (3) would be a better model for most DE genes, global analysis has better power. Our small sample size has precluded our ability to rule out the contributions of several important covariates that may affect gene expression and the potential effect of diagnosis. These include potential effects of pharmacotherapy, substance use, and additional neurological and psychiatric diagnoses. It is worth noting that in a previous study of these same subjects, no impact of major depressive disorder (MDD) diagnosis or antidepressant, benzodiazepine, or tobacco use was observed on expression of synaptic transcripts in the OFC and striatum^36^. Third, although these data are broadly consistent with our previous report of downregulation of a small targeted set of glutamatergic transcripts assessed via qPCR across OFC and striatal brain regions in these same OCD subjects^36^, here we did not observe significant DEG in the OFC alone. Notably, on a gene-by-gene basis, the normalized qPCR expression from^36^ and the RNAseq gene expression levels reported here were significantly positively correlated as expected (*r* = 0.340, *p* < 0.0001, Table S11). It is therefore likely that there are changes in transcript levels in the OFC of our OCD subjects relative to unaffected comparison subjects, but our small sample size and multiple comparison correction limited our ability to detect them. This interpretation is bolstered by our global analysis demonstrating many more significant differentially expressed transcripts in the global condition (OFC and striatum) than in the striatum alone. Fourth, using the metric of cell type fractions, we cannot currently distinguish changes in cellular and anatomical structure (e.g., reduced medium spiny neuron number) from reductions in synaptic transcripts expressed within those cells. Future experiments will seek to differentiate these possibilities. Despite these limitations, the utility of performing unbiased transcriptomic analysis is clear as it provides a more complete picture of dysfunction across regions and allows for the identification of novel transcripts and signaling pathways that would not otherwise be detected. Furthermore, we demonstrate striking consistency with a previous RNA-sequencing study in disrupted molecular pathways and specific striatal transcripts identified as DEGs, suggesting potential common molecular mechanisms underlying OCD pathogenesis.

Here we conducted the first unbiased transcriptomic analysis of post-mortem tissue from OFC and striatum in OCD and unaffected comparison subjects. We found significantly lower expression of transcripts associated with synaptic neurotransmission across all regions and determined that these changes may be strongest in medium spiny neurons in the caudate nucleus and nucleus accumbens. Further examination of these data could help identify novel treatment targets for OCD and related disorders and will serve as a foundation for future larger studies.

## Supplementary information

Supplemental Material 1

Supplemental Figure 1

Supplemental Figure 2

Supplemental Figure 3

Supplemental Figure 4

Supplemental Figure 5

Supplemental Figure 6

Supplemental Table 1

Supplemental Table 2

Supplemental Table 3

Supplemental Table 4

Supplemental Table 5

Supplemental Table 6

Supplemental Table 7

Supplemental Table 8

Supplemental Table 9

Supplemental Table 10

Supplemental Table 11
