# Supplementary material for: Transcriptome alterations are enriched for synapse-associated genes in the striatum of subjects with obsessive-compulsive disorder
Source: Transl Psychiatry. 2021 Mar 15;11:171. doi: 10.1038/s41398-021-01290-1 (PMC7961029; doi:10.1038/s41398-021-01290-1)
Supplement: Supplementary file 1 — Supplemental Material 1 [file 41398_2021_1290_MOESM1_ESM.docx]

**SUPPLEMENTAL MATERIAL**

**METHODS**

*Tissue Collection & RNA Extraction:* Standardized amounts (50 mm3) of gray matter were collected via dissection of sections cut on cryostat from four separate brain regions identified cytoarchitectonically: medial orbitofrontal cortex (mOFC, BA11), lateral orbitofrontal cortex (lOFC, BA47), head of the caudate nucleus, and nucleus accumbens core. RNA was extracted using an RNeasy Plus Mini kit (QIAGEN, Valencia, CA) as previously described [1]. RNA quality was assessed using RNA ScreenTape Analysis (Agilent Technologies, Santa Clara, CA) and RNA concentration was estimated using a Qubit Fluorometer (ThermoFisher Scientific, Waltham, MA).

*RNA sequencing:* Messenger RNA (mRNA) sequencing was performed using the Illumina NextSeq 500 platform (Illumina Inc, San Diego, CA). Complementary DNA (cDNA) libraries were generated using TruSeq Stranded mRNA Library Preparation kits (Illumina Inc, San Diego, CA) in which poly-adenylated mRNA molecules were purified, fragmented, and then reverse transcribed into first and second cDNA strands. Single-end mRNA sequencing was then performed at a targeted depth of 40 million reads per sample. Reads were aligned to human genome reference sequence GRCh38.p10 and annotated via Ensemble Archive Release v91; the number of reads that mapped (or did not map) to the reference sequence is depicted in Supplemental Figure 1. Quality control (QC) was conducted using CLC Genomics (QIAGEN) and raw read counts were exported for downstream analyses.

*Differential Expression:* To identify drivers of variation in gene expression, the relationship between gene expression values and covariates – diagnosis, sex, age, post-mortem interval, pH, RNA integrity number, or brain region – were assessed using generalized linear regression [2]. Briefly, following Fromer, Roussos [3], we sought a parsimonious model consisting of covariates that predicted each gene’s expression. To accomplish this goal, we implemented a stepwise forward procedure, beginning with diagnosis and sex as the first predictors in the model. Next, the covariate that maximized the number of genes showing improved fit between the covariate and gene expression, as judged by Bayesian information criterion (BIC), was added to this model. This process of sequentially adding covariates to the model was repeated until only a minority of the gene expression values (<5%) were improved by the next covariate (Supplemental Table 1). Although the relatively small sample size limits efficacy of selecting covariates for predicting variation in gene expression, we believe this process, which includes only covariates that statistically affect transcript expression, is sufficient to obtain a robust model.

To identify genes differentially expressed between OCD subjects and unaffected comparison subjects, either across brain regions or within a single region, we used the covariate model described above. Expression counts were transformed into log2CPM and fitted to covariates using limma. For each gene, the standard deviation of expression was estimated using empirical Bayes techniques to obtain voom precision weights, which were then used to estimate differential expression in this weighted linear regression context [4-6]. Significance of each contrast was determined using Bayes factors, assuming that 1% of genes were expected to be differentially expressed. When analyzing the regions simultaneously, we used the average correlation among regions in gene expression, estimated by limma, to account for repeated observations within subject. Region-specific effects were then estimated simultaneously for all regions, after which significance was judged for appropriate contrasts (FDR-adjusted p-value was set at α=0.05).

*Data Quality Control:* On average, 98.5% of reads were mapped for each sample and the number of mapped reads did not differ significantly between OCD subjects and unaffected comparison subjects (t=1.6; df=14; p=0.14; Supplemental Figure 1). Sequence reads for the four brain regions of the 16 subjects were mapped to 58,243 transcripts, which were further reduced to 18,993 expressed RefSeq genes (v.2015-01). Of those, genes were retained that had at least 1 count per million (CPM) in at least half of the samples for at least one of the four brain regions; and if, over all subjects and brain regions, gene j’s coefficient of variation CVj<T, where T is defined as the mean of CVj over all j plus 3 standard deviations of CV. This resulted in 14,211 genes whose expression passed QC.

To evaluate the extent to which gene expression was distinct for the four brain regions, pairwise consensus correlations were obtained from the limma package in R (v.3.3.19; [2, 6]) and visualized using a heatplot (pheatmap package in R) over all subjects. Pairwise consensus correlations over genes were high between BA11 and BA47 (0.71) compared to other pairwise correlations (0.16-0.25; Supplemental Figure 2). Thus, data for BA11 and BA47 were treated as from a single brain region termed "OFC" by averaging expression, per gene and subject, over BA11 and BA47. In this process, however, we noted one male OCD subject for whom the BA47 and nucleus accumbens samples had likely been inadvertently switched. Thus, this subject was removed from further analysis. After removal, seven OCD subjects and eight unaffected comparison subjects remained. After re-running the QC steps based on the new data, specifically over three brain regions and with one subject removed, there were 14,184 genes remaining for analyses for all brain regions and 13,623, 13,889, and 13,756 for OFC, caudate, and NAcc, respectively.

*Gene Set Enrichment:* To investigate whether differentially expressed genes between OCD subjects and unaffected comparison subjects were enriched for biologically relevant gene-set pathways, we used the Gene Set Enrichment Analysis (GSEA) platform [7]. This platform contains various molecular signature databases (MSigDB, v7.0; [8]), including curated gene sets that are publicly available and biologically relevant. Enrichment was evaluated on the differentially expressed genes from the analysis of all brain regions together and per individual brain region. Within the MSigDB, we focused on the following general gene sets (excluding sets that were curated for specific purposes): Hallmark [50 sets]; the C2 curation (sub-databases included canonical [2,199 sets], KEGG [186 sets], and REACTOME [1,499 sets]); and the C5 Gene Ontology (GO) curation (sub-databases included biological process [7,350 sets], cellular component [1,001 sets], and molecular function [1,645 sets]). For each gene set containing at least 30 genes, differentially expressed genes were analyzed for enrichment using Fisher's exact test and “significance” judged by FDR (for sets with enrichment odds ratio exceeding one).

We determined co-occurrence for the GO gene sets that were significantly enriched in OCD in the global analysis. Genes were defined as co-occurring if present in more than one gene set. A distance value was computed per gene set pair, defined as the fraction of overlapping genes divided by the number of unique genes [9]. A network was computed using the force-directed graph drawing algorithm directed by Fruchterman-Reingold (Figure 2) [10]. Hierarchical clustering was performed on the computed distance values using the Ward D2 method [11]; within the networks, four sub-networks were now distinguishable (Figure 2).

*Cell Type Composition:* The composition of the tissue samples in terms of broad cell type fractions–excitatory neurons, medium spiny neurons (also known as spiny projection neurons), interneurons, astrocytes, oligodendrocytes, ependymal cells, immune cells, and vascular cells – was estimated using deconvolution methods. The principle behind this method is that the bulk expression of a gene for a tissue sample, here measured using RNAseq, is a convolution of the number of cells of each type comprising the sample and the average expression of the gene within the cells of each type. Although the estimates from deconvolution more closely map onto “expression fractions”, we will continue to follow convention and refer to these as “cell type fractions”. To identify a specific feature of this convolution, namely the fraction of each cell type, we utilized a reference transcriptome of single cell-based RNAseq data obtained from mouse cortex and striatum and containing 62,155 cells of various cell types [12]. This reference database was used to derive a “signature matrix” (genes by cell types), which was generated by averaging the observed expression of the top 50 marker genes per cell type over 62,155 cells belonging to the general cell types described above (following [13]). While this reference database was generated using single-cell sequencing data from the mouse, we also demonstrate that using a reference from human snRNA of the cortex and caudate [14] produces very similar results (Supplemental Figure 4). Finally, using the observed gene expression patterns in the region and the signature matrix, non-negative least squares deconvolution estimated the cell type fractions for each brain region and subject in our dataset. Cell type expression profiles of OCD subjects were compared to unaffected comparison subjects using a linear model, adjusting for brain region, sex, age, PMI, pH, and RIN, for the combined brain regions and each region separately.

**LITERATURE CITED**

1. Piantadosi, S.C., et al., *Lower excitatory synaptic gene expression in orbitofrontal cortex and striatum in an initial study of subjects with obsessive compulsive disorder.* Mol Psychiatry, 2019.

2. Bates, D., et al., *Fitting Linear Mixed-Effects Models Using lme4.* 2015, 2015. **67**(1): p. 48 %J Journal of Statistical Software.

3. Fromer, M., et al., *Gene expression elucidates functional impact of polygenic risk for schizophrenia.* Nat Neurosci, 2016. **19**(11): p. 1442-1453.

4. Law, C.W., et al., *voom: Precision weights unlock linear model analysis tools for RNA-seq read counts.* Genome Biol, 2014. **15**(2): p. R29.

5. Phipson, B., et al., *Robust Hyperparameter Estimation Protects against Hypervariable Genes and Improves Power to Detect Differential Expression.* Ann Appl Stat, 2016. **10**(2): p. 946-963.

6. Ritchie, M.E., et al., *limma powers differential expression analyses for RNA-sequencing and microarray studies.* Nucleic Acids Res, 2015. **43**(7): p. e47.

7. Subramanian, A., et al., *Gene set enrichment analysis: a knowledge-based approach for interpreting genome-wide expression profiles.* Proc Natl Acad Sci U S A, 2005. **102**(43): p. 15545-50.

8. Liberzon, A., et al., *Molecular signatures database (MSigDB) 3.0.* Bioinformatics, 2011. **27**(12): p. 1739-40.

9. Ruckdeschel, P., et al., *S4 Classes for Distributions.* R News, 2006. **6(2)**: p. 2-6.

10. Fruchterman, T.M.J. and E.M. Reingold, *Graph drawing by force-directed placement.* 1991. **21**(11): p. 1129-1164.

11. Müllner, D., *fastcluster: Fast Hierarchical, Agglomerative Clustering Routines for R and Python.* 2013, 2013. **53**(9): p. 18 %J Journal of Statistical Software.

12. Zeisel, A., et al., *Molecular Architecture of the Mouse Nervous System.* Cell, 2018. **174**(4): p. 999-1014 e22.

13. Wang, J., B. Devlin, and K. Roeder, *Using multiple measurements of tissue to estimate subject- and cell-type-specific gene expression.* Bioinformatics, 2020. **36**(3): p. 782-788.

14. Khrameeva, E., et al., *Single-cell-resolution transcriptome map of human, chimpanzee, bonobo, and macaque brains.* Genome Res, 2020. **30**(5): p. 776-789.

**SUPPLEMENTAL TABLE 1.** Number of genes for which the Bayesian information criterion (BIC) improved during model selection.

**SUPPLEMENTAL TABLE 2.** Analysis of differential gene expression for combined brain regions and for each brain region separately. FC, fold change; CPM, counts per million; t, Student's t-statistic; FDR, false discovery rate.

**SUPPLEMENTAL TABLE 3.** Gene sets enriched for differentially expressed genes between OCD subjects and unaffected comparison subjects. Gene sets enriched for differentially expressed genes were identified using Fisher's exact test. Gene sets containing at least 30 genes were considered significant if the enrichment odds ratio was >1.0 and the False Discovery Rate (FDR) q-value was <0.05. A summary of the enriched gene sets is provided in Supplemental Table 3a. Enriched gene sets were identified that contained either upregulated or downregulated genes in OCD subjects versus unaffected comparison subjects (Supplemental Table 3b); that contained only upregulated genes (Supplemental Table 3c); or that contained only downregulated genes (Supplemental Table 3d). DEG: differentially expressed genes; OR: odds ratio; P: probability.

**SUPPLEMENTAL TABLE 4.** Cell-type fraction estimations in post-mortem brain tissue from OCD subjects and unaffected comparison subjects.

**SUPPLEMENTAL TABLE 5.** Region-specific differences of cell type fractions between OCD subjects and unaffected comparison subjects. Listed numbers = p-values (uncorrected for multiple comparisons). As expected, there were zero estimated fractions for medium spiny neurons in the orbitofrontal cortex and for excitatory neurons in caudate and nucleus accumbens; thus, the corresponding p-values are marked NA.

**SUPPLEMENTAL TABLE 6.** Cell-type fraction estimations in post-mortem brain tissue from OCD subjects and unaffected comparison subjects. Analysis conducted using human snRNA-seq data from [44].

**SUPPLEMENTAL TABLE 7.** Region-specific differences of cell type fractions between OCD subjects and unaffected comparison subjects using human snRNA-seq data from [44]. Listed numbers = p-values (uncorrected for multiple comparisons). As expected, there were zero estimated fractions for medium spiny neurons in the orbitofrontal cortex and for excitatory neurons in caudate; thus, the corresponding p-values are marked NA.

**SUPPLEMENTAL TABLE 8.** Characteristics of current study and those of Lisboa et al. and results from comparison between studies. Results from fitting a mixture model over a standard Beta(a,1) distribution and a Uniform(0,1) distribution, with a mixing parameter determining the fraction of observations coming from the Beta distribution. The model was fitted to the data by maximum likelihood using 'fitBumModel' in R library BioNet. --: decreased expression; ++: increased expression.--: Decreased expression; ++: Increased expression FC, fold change; FDR, false discovery rate; NAcc, nucleus accumbens core.

**SUPPLEMENTAL TABLE 9.** Comparison of DEG from Lisboa et al. [37] and the present study.

**SUPPLEMENTAL TABLE 10.** Gene sets enriched for genes congruent between Lisboa et al. [37] and present study. GSterm, gene set term; GEgenes, number of genes in gene set; DEgenes, differentially expressed gene count; OR, odds ratio.

**SUPPLEMENTAL TABLE 11.** Comparison of previous qPCR expression values Piantadosi et al. [36] and present study. Values represent Pearson correlation coefficients. Empty cells indicate transcripts below detection via qPCR.

**SUPPLEMENTAL FIGURE 1.** RNA sequencing data read counts. The counts of the number of RNA sequencing reads that mapped (blue) or did not map (red) to the human reference sequence are shown per subject per brain region. X-axis indicates subject number.

**SUPPLEMENTAL FIGURE 2.** Hierarchical clustering and heatmap of RNA expression obtained from four post-mortem brain regions in seven OCD subjects and eight unaffected comparison subjects. The similarity between each sample pair was computed using the Euclidean distance of the log_2_(count per million) reads. Two main clusters were observed containing the Brodmann areas (pink and gray boxes indicated at the top of the figure) and the striatal regions (blue and orange boxes indicated at the top of the figure).

**SUPPLEMENTAL FIGURE 3.** Volcano plot of the differentially expressed genes from cortical regions in OCD subjects. The y-axis represents the (-log_10_P-value) and the x-axis represents the gene expression log_2_fold change. Vertical dashed lines (±0.26 log_2_ fold change) indicate expression difference where upregulated genes are positive and downregulated genes are negative. The horizontal line demarcates significantly different gene expression differences between OCD subjects and unaffected comparison subjects (false discovery rate q-value <0.05).

**SUPPLEMENTAL FIGURE 4.** Cell type fractions in OCD subjects and unaffected comparison subjects. Cell type fractions were determined across all brain regions for OCD subjects and unaffected comparison subjects separately. Gene expression patterns in single-cell data obtained from human snRNA-seq data [44] were used to estimate the fraction of seven broadly defined cell types: astrocytes, excitatory neurons, immune cells, interneurons, oligodendrocytes, oligodendrocyte precursor cells, and medium spiny neurons. Linear regression was used to compare OCD subjects (purple) and unaffected comparison subjects (grey) in each of the eight cell fractions. Boxplots are displayed, where ** indicates p<0.01 and * indicates p<0.05 (uncorrected for multiple comparisons). The mean cell type fractions were computed based on all four brain regions; however, when the cell type did not exist in the brain region, it was not included in the calculation. Thus, the mean cell type fraction for the excitatory neurons did not include the caudate, and the mean cell type fraction for spiny projection neurons was based on the caudate and nucleus accumbens regions.

**SUPPLEMENTAL FIGURE 5.** Distribution of p-values for DEG from our study that were also reported to be DEG in the Lisboa et al. [37]. The red line traces the predicted value for the estimated standardized Beta distribution, with estimated parameter value “a” shown as the horizontal blue line. Inset: comparison of log-fold change for our study (Piantadosi et al.) and the Lisboa et al. study for genes reported to be DEG in Lisboa et al.

**SUPPLEMENTAL FIGURE 6.** Tree map synopsis of Gene Ontology GO terms from gene set enrichment analysis. The tree map was produced by REVIGO. Genes used for enrichment analysis are those reported to be DEG in the Lisboa study and showing strong agreement (FDR ≤ 0.20 and congruent in direction) in our study (263 total genes). Enriched GO terms are reported in Supplementary Table 10.
