# Supplementary figures and images for: Transcriptome alterations are enriched for synapse-associated genes in the striatum of subjects with obsessive-compulsive disorder
Source: Transl Psychiatry. 2021 Mar 15;11:171. doi: 10.1038/s41398-021-01290-1 (PMC7961029; doi:10.1038/s41398-021-01290-1)

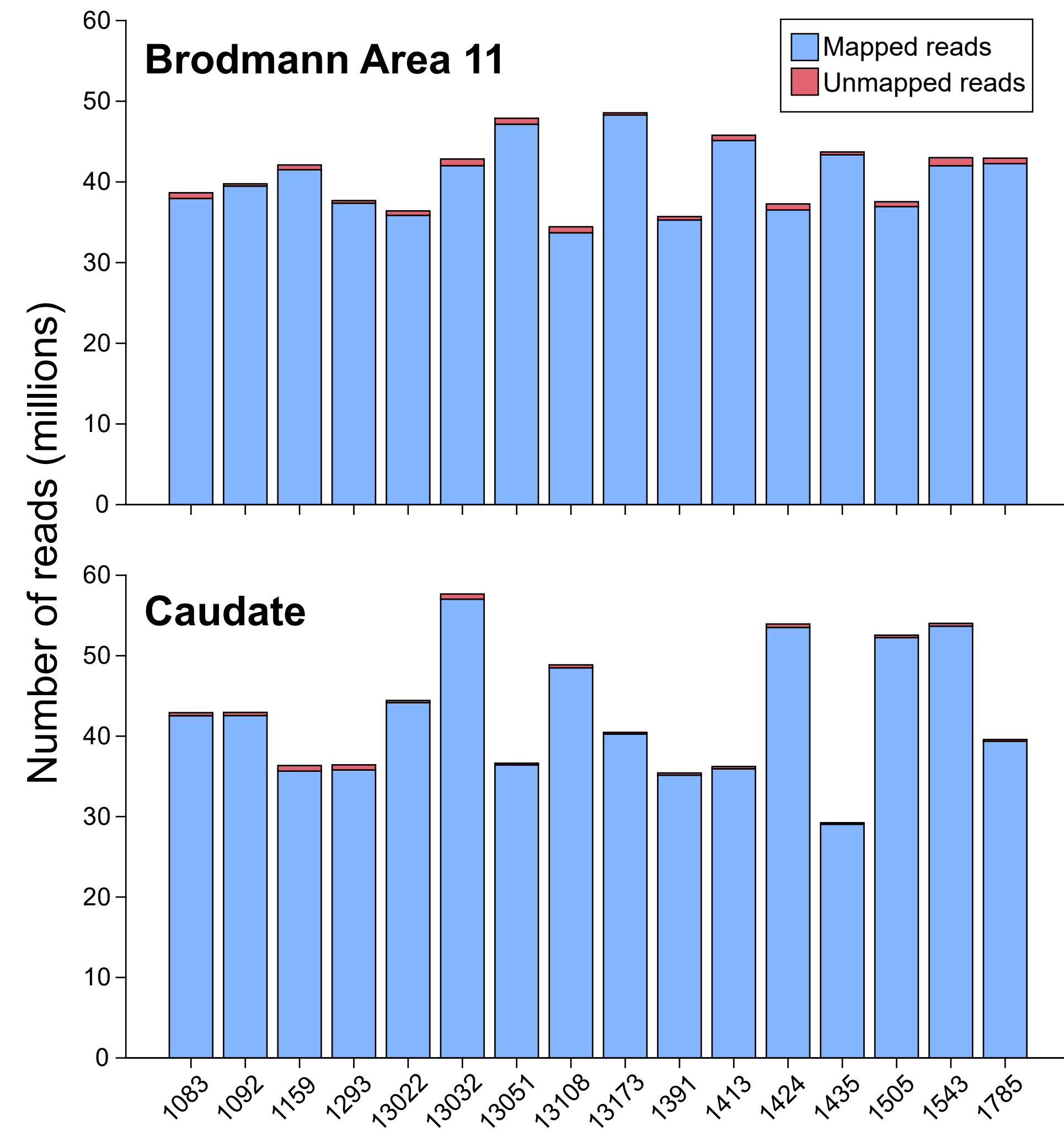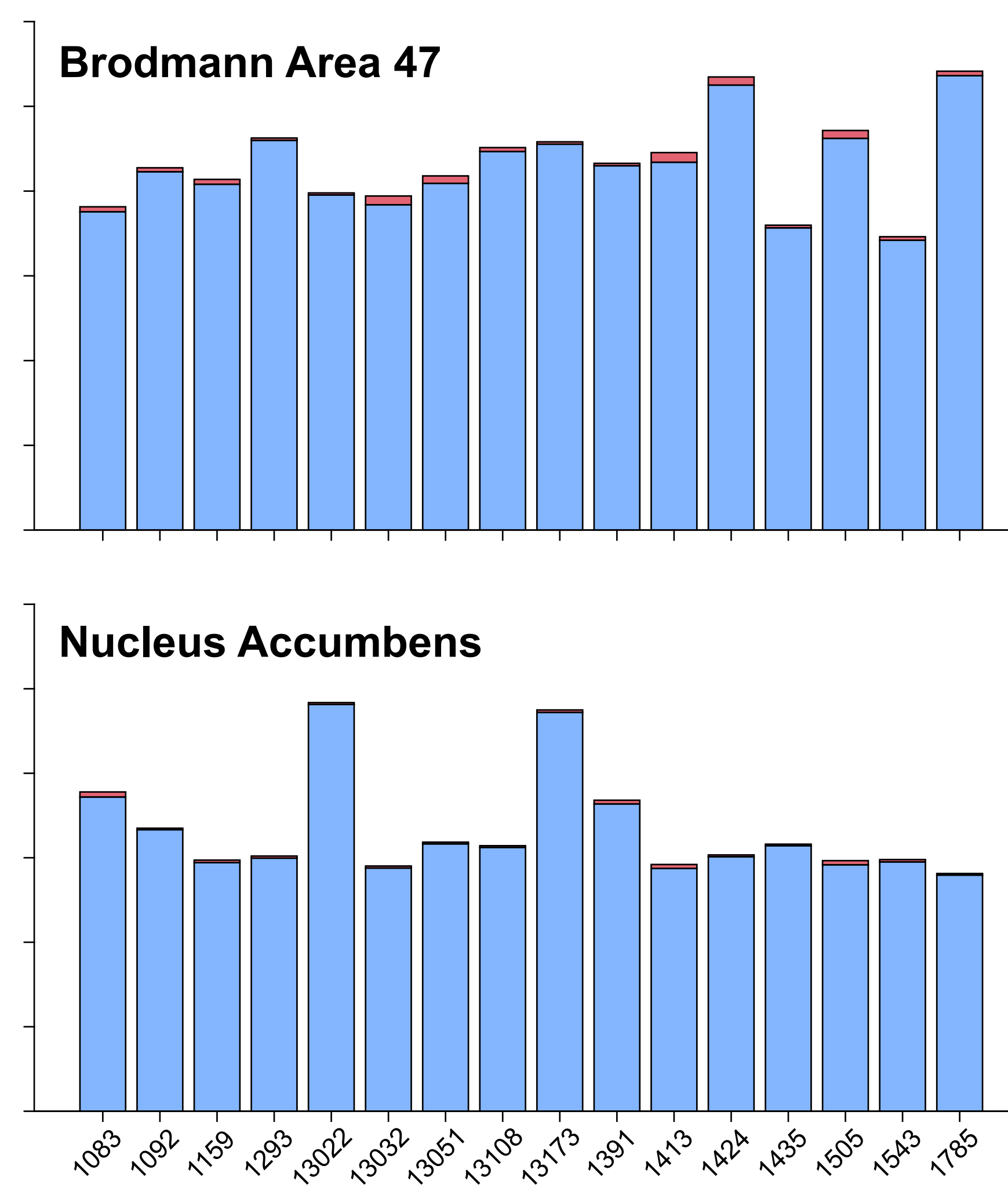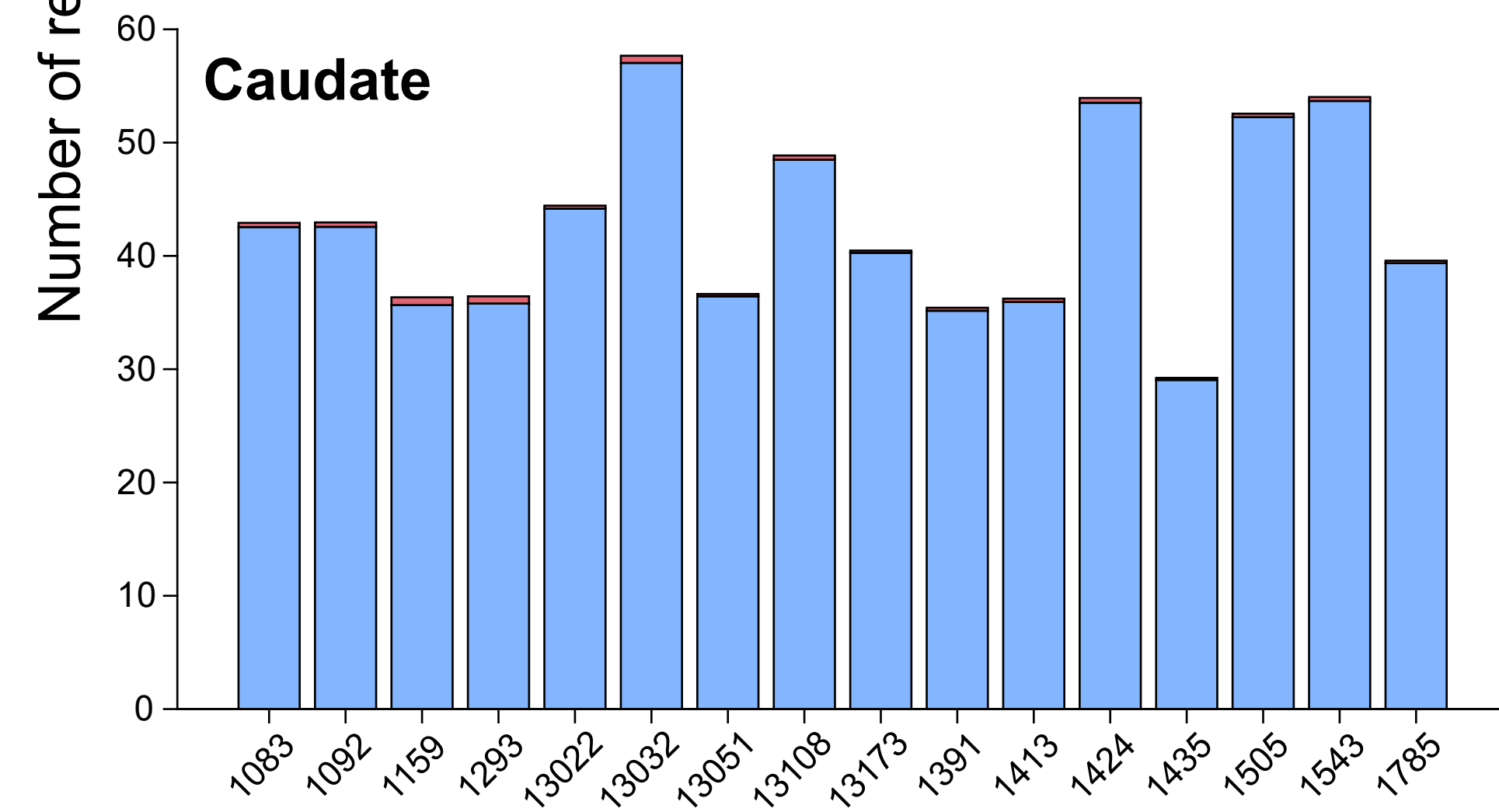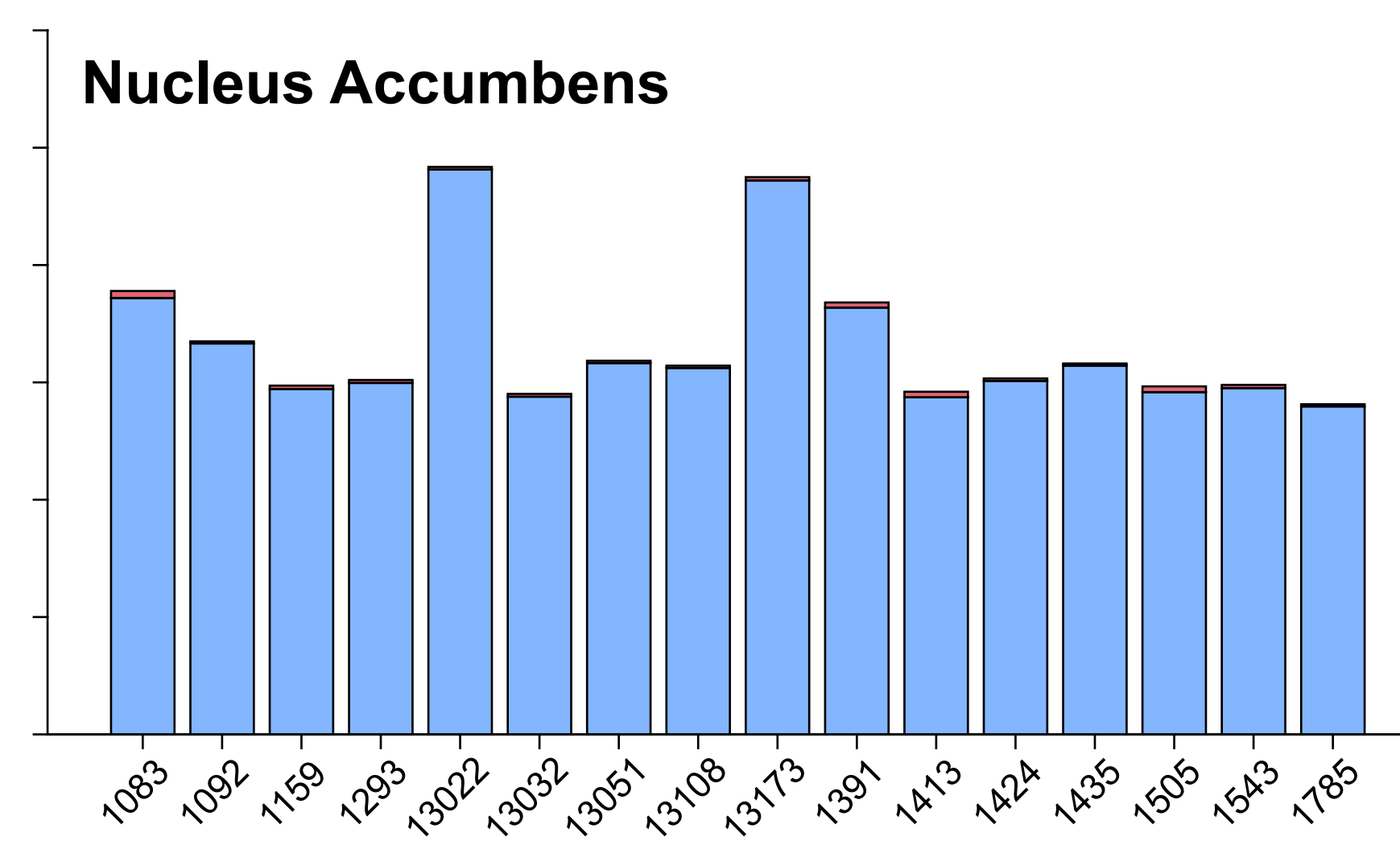

Supplemental Figure 1

Supplement: Supplementary file 2 — Supplemental Figure 1 [file 41398_2021_1290_MOESM2_ESM.pdf]

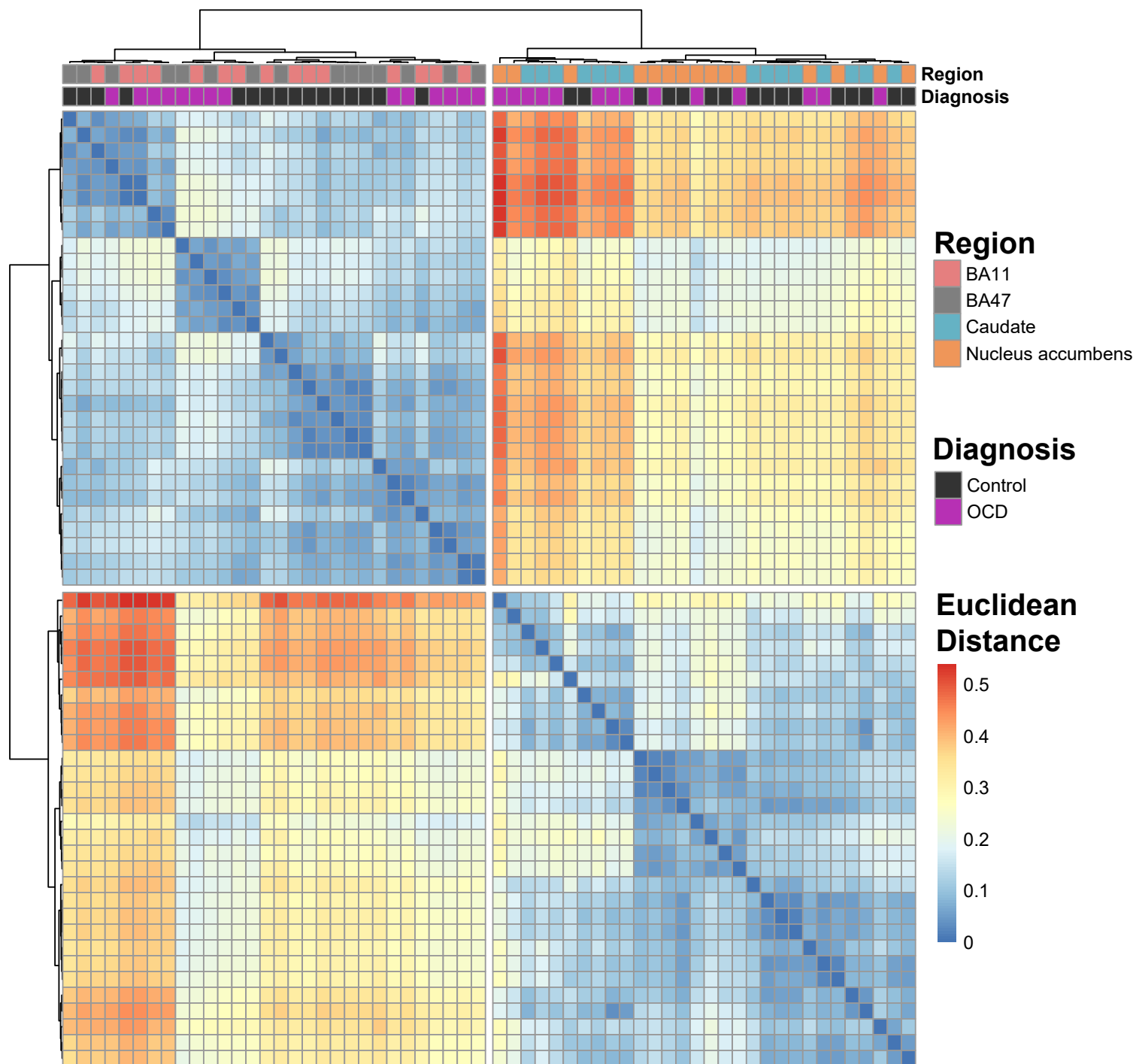

Supplemental Figure 2

Supplement: Supplementary file 3 — Supplemental Figure 2 [file 41398_2021_1290_MOESM3_ESM.pdf]

# Orbitofrontal Cortex

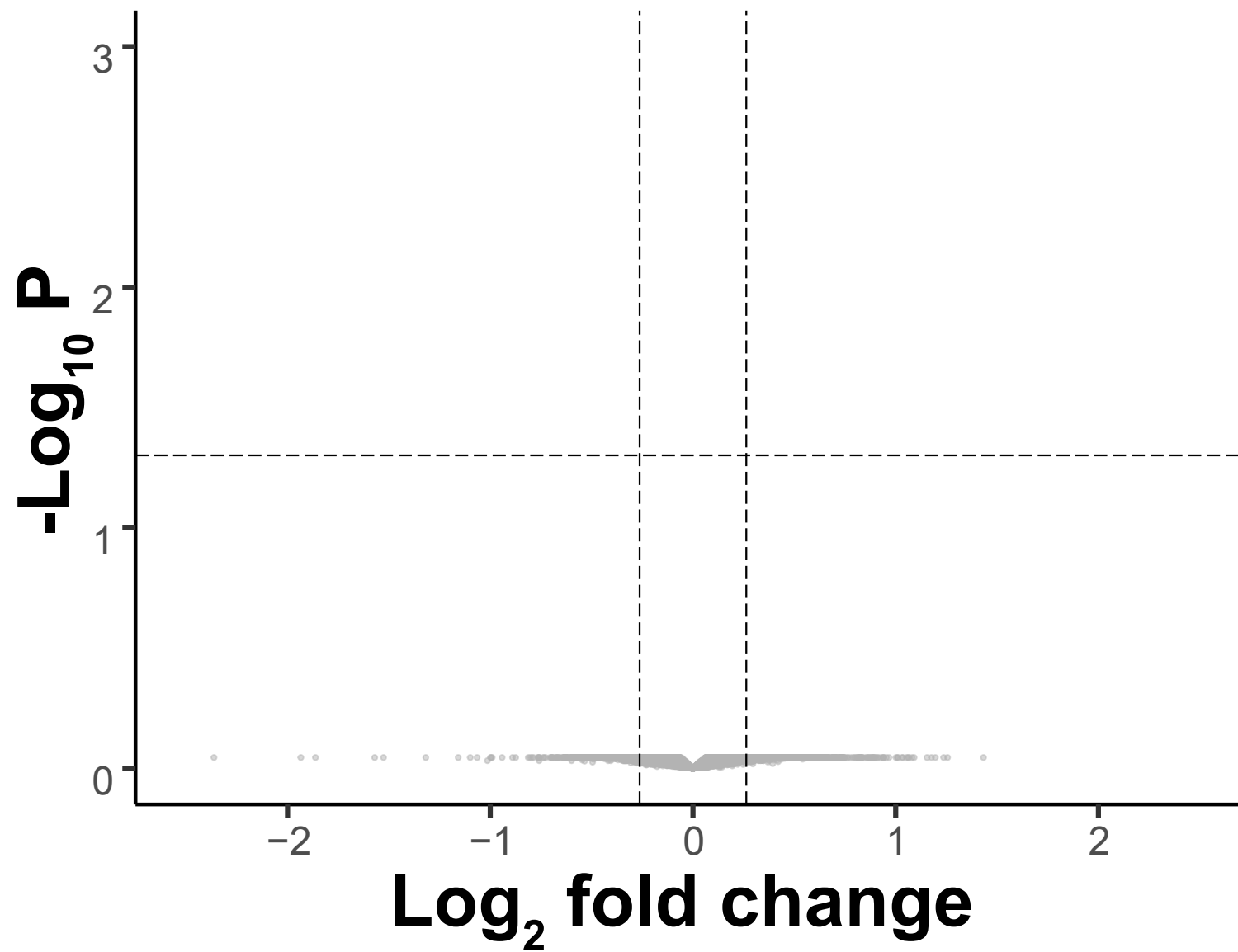

Supplemental Figure 3

Supplement: Supplementary file 4 — Supplemental Figure 3 [file 41398_2021_1290_MOESM4_ESM.pdf]

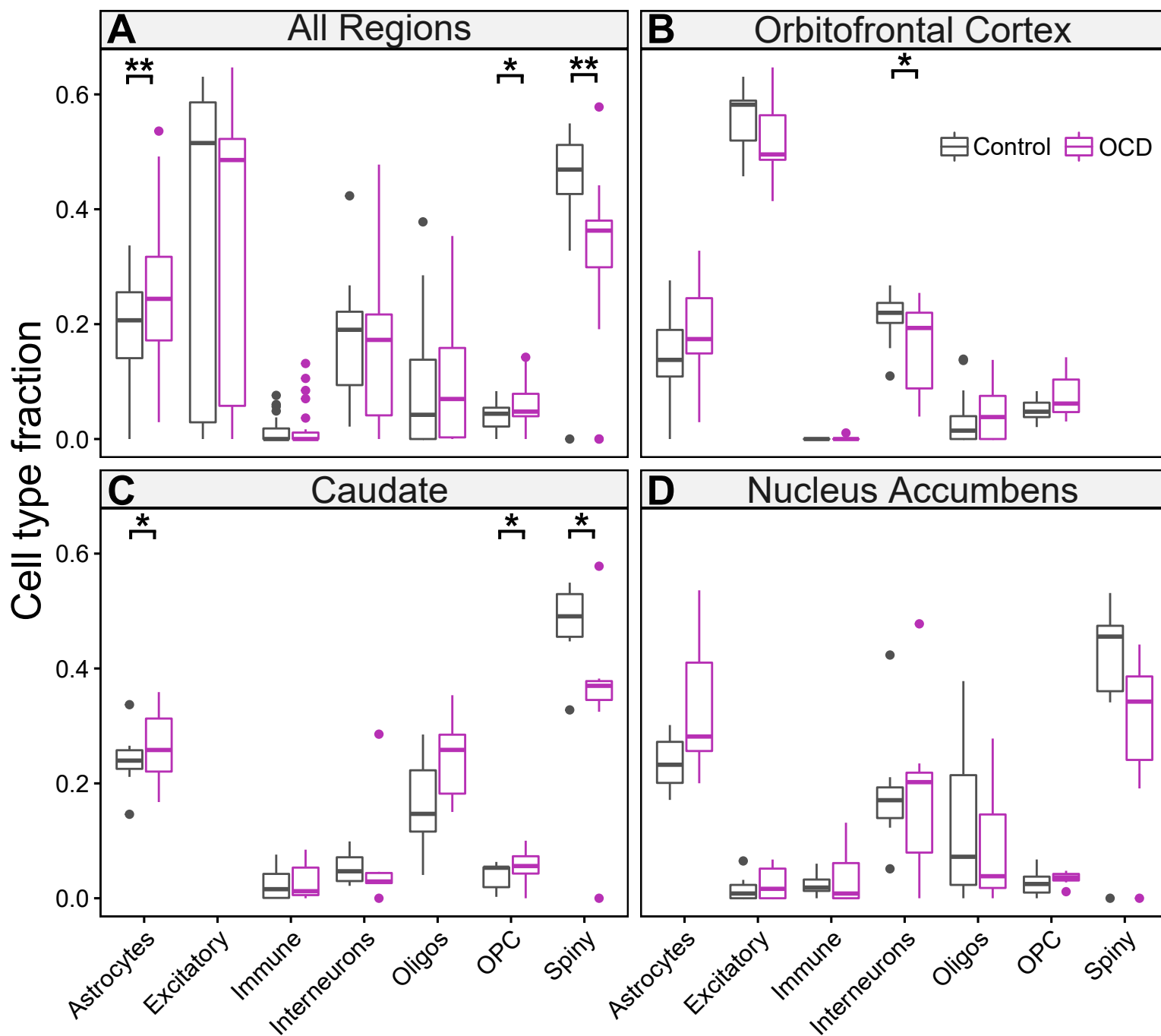

Supplemental Figure 4

Supplement: Supplementary file 5 — Supplemental Figure 4 [file 41398_2021_1290_MOESM5_ESM.pdf]

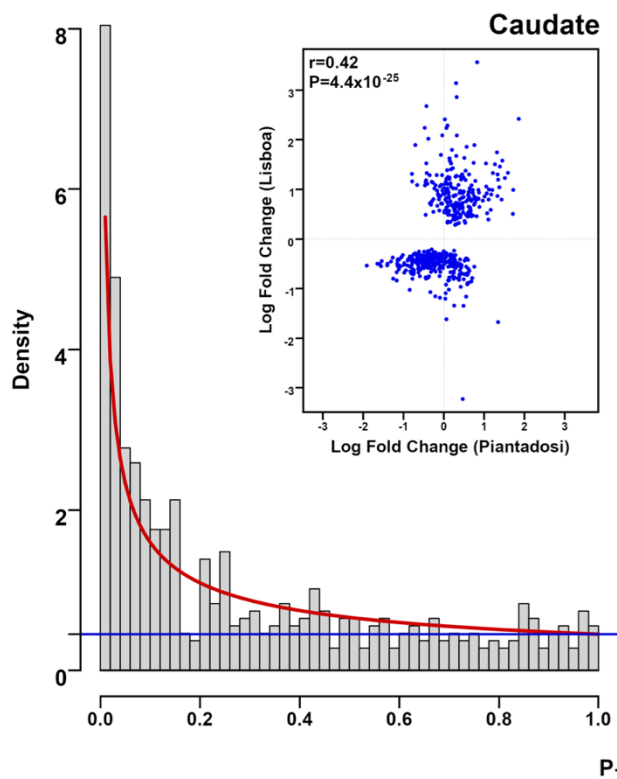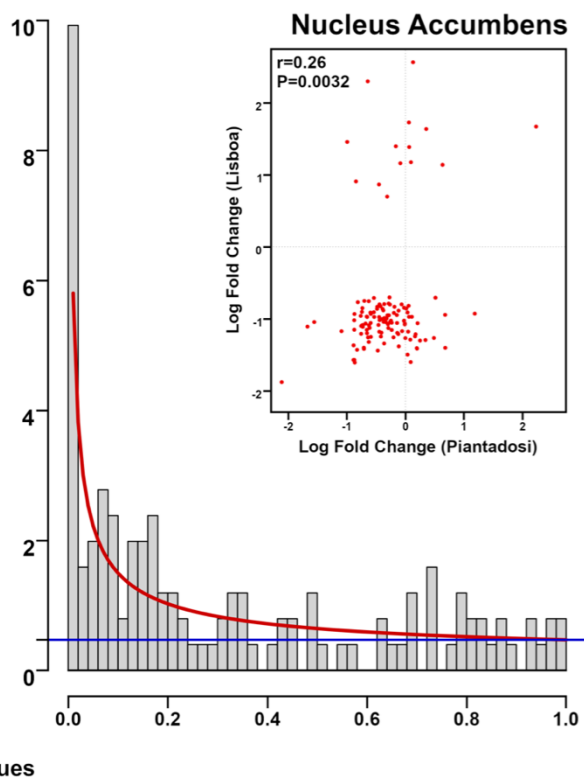

**Supplemental Figure 5**

Supplement: Supplementary file 6 — Supplemental Figure 5 [file 41398_2021_1290_MOESM6_ESM.pdf]

REVIGO Gene Ontology treemap

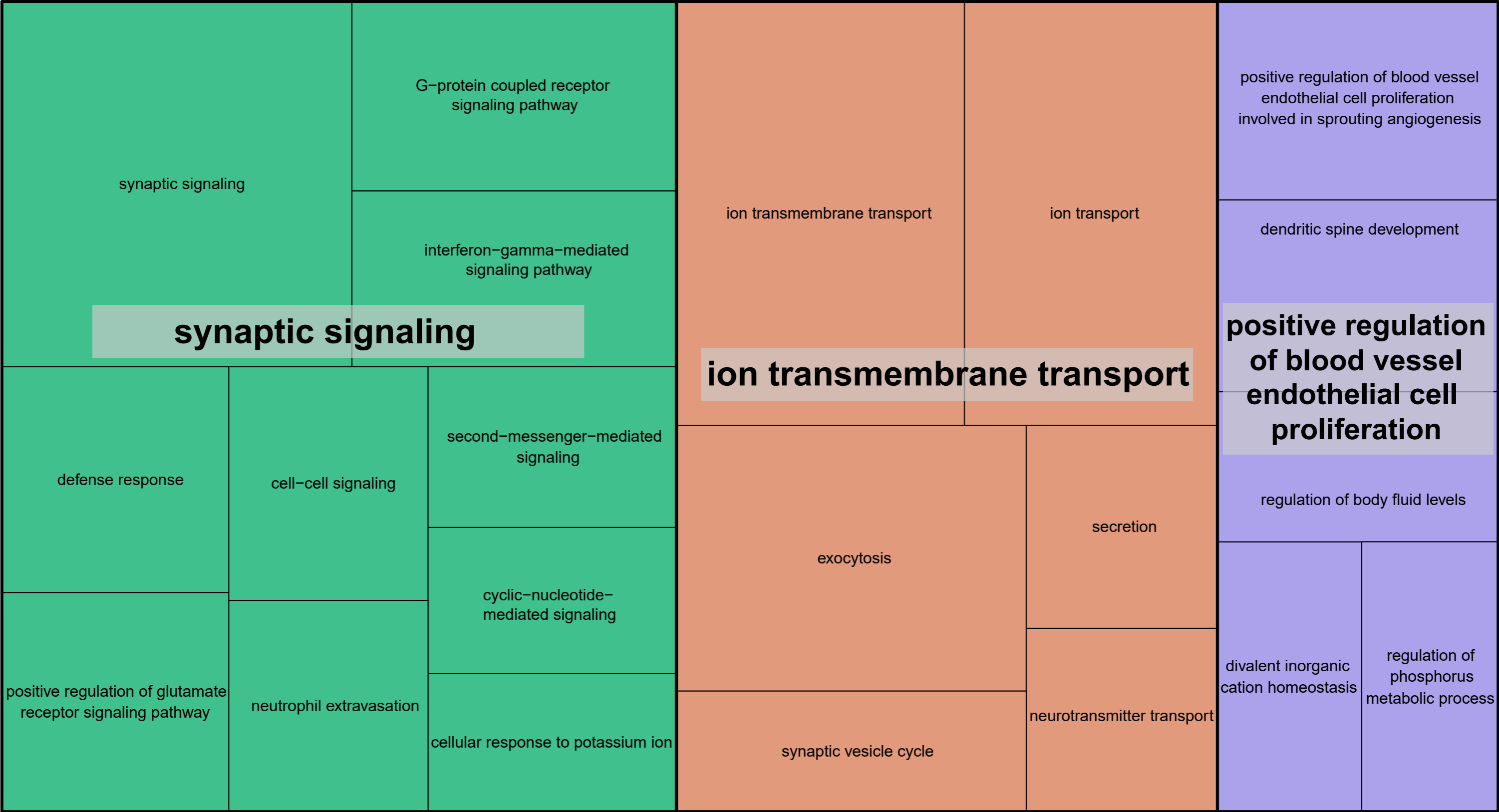

Supplemental Figure 6

Supplement: Supplementary file 7 — Supplemental Figure 6 [file 41398_2021_1290_MOESM7_ESM.pdf]
